# Correction: Tomasino et al. Diversity and Hydrocarbon-Degrading Potential of Deep-Sea Microbial Community from the Mid-Atlantic Ridge, South of the Azores (North Atlantic Ocean). *Microorganisms* 2021, *9*, 2389

**DOI:** 10.3390/microorganisms10050988

**Published:** 2022-05-09

**Authors:** Maria Paola Tomasino, Mariana Aparício, Inês Ribeiro, Filipa Santos, Miguel Caetano, C. Marisa R. Almeida, Maria de Fátima Carvalho, Ana P. Mucha

**Affiliations:** 1 CIIMAR—Interdisciplinary Centre of Marine and Environmental Research, University of Porto, Terminal de Cruzeiros do Porto de Leixões, Avenida General Norton de Matos, s/n, 4450-208 Matosinhos, Portugal; mariana_aparicio_30@hotmail.com (M.A.); inesfcribeiro@gmail.com (I.R.); pipa.santos2@gmail.com (F.S.); mcaetano@ipma.pt (M.C.); calmeida@ciimar.up.pt (C.M.R.A.); mcarvalho@ciimar.up.pt (M.d.F.C.); amucha@ciimar.up.pt (A.P.M.); 2 Instituto Português do Mar e da Atmosfera, I.P. Avenida de Brasília, 1449-006 Lisboa, Portugal; 3 School of Medicine and Biomedical Sciences (ICBAS), University of Porto, Rua de Jorge Viterbo Ferreira 228, 4050-313 Porto, Portugal; 4 Faculty of Sciences, University of Porto, Rua do Campo Alegre 790, 4150-171 Porto, Portugal

The authors wish to make the following corrections to this paper [1]:

After the publication of the manuscript, the authors recognized that one reference in the discussion session (in the main text) was wrong and should be replaced with a new one. The changes are provided below. 

## Changes in Discussion

Furthermore, Potts et al. [58] in a recent work reported differences in hydrocarbons removal between shallow and deep sediment consortia (50–58% and 33–42%, respectively), highlighting the role of consortium origin.

## Change in the Reference

58. Potts, L.D.; Calderon, L.J.P.; Gontikaki, E.; Keith, L.; Gubry-Rangin, C.; Anderson, J.; Witte, U. Effect of spatial origin and hydrocarbon composition on bacterial consortia community structure and hydrocarbon biodegradation rates. *FEMS Microbiol. Ecol.* **2018**, *94*, 127.

The authors apologize for any inconvenience caused and state that the scientific conclusions are unaffected. The original publication has also been updated.
