# Supplementary material for: Diversity and Hydrocarbon-Degrading Potential of Deep-Sea Microbial Community from the Mid-Atlantic Ridge, South of the Azores (North Atlantic Ocean)
Source: Microorganisms. 2021 Nov 19;9(11):2389. doi: 10.3390/microorganisms9112389 (PMC8620031; doi:10.3390/microorganisms9112389)
Supplement: Supplementary file 1 [file microorganisms-09-02389-s001.zip › microorganisms-1401897-supplementary.pdf]

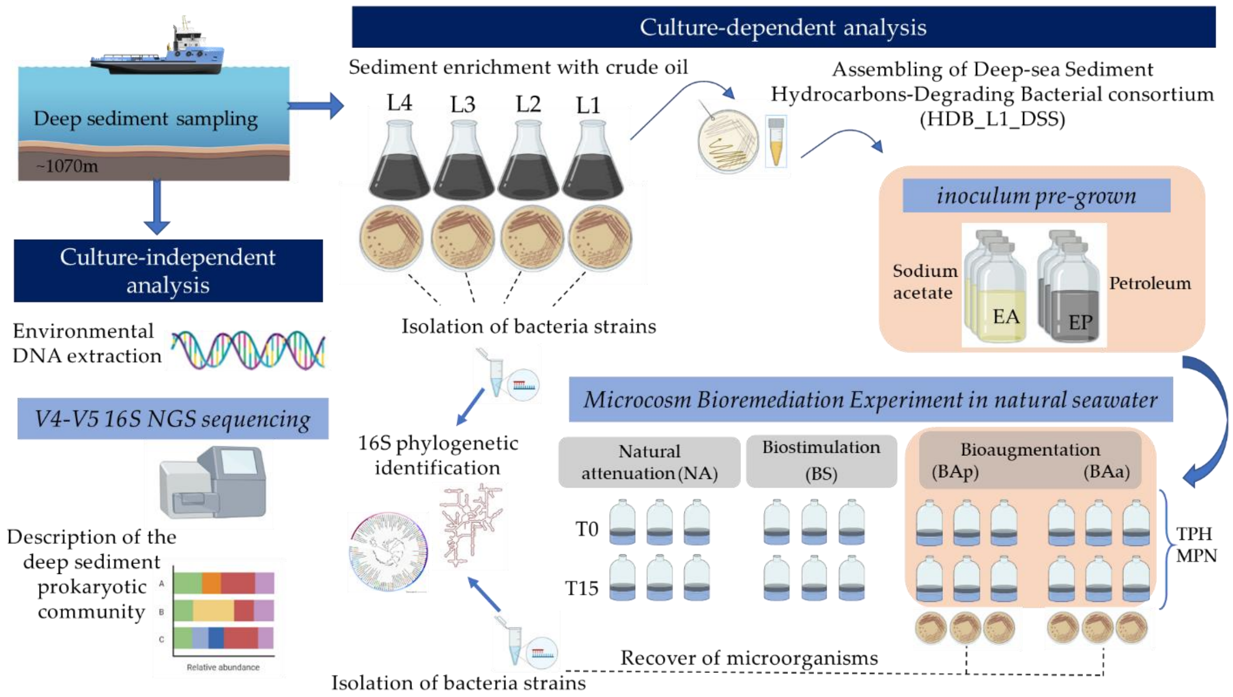

Figure S1. Workflow.

Table S1. Summary statistics of 16S rRNA gene amplicon sequencing.

Sample ID	# Sequences	# OTUs[a] (%)	#Classified (%)	# No Relative (%)	# Rejected (%)
**L1**	46,874	10,320 (22.02)	42,879 (91.48)	531 (1.13)	3,464 (7.39)
**L2**	31,328	4,788 (15.28)	28,545 (91.12)	352 (1.12)	2,431 (7.76)
**L3**	86,322	17,180 (19.90)	71,911 (83.31)	989 (1.15)	13,422 (15.55)
**L4**	74,883	13,785 (18.41)	71,080 (94.92)	727 (0.97)	3,076 (4.11)

[a]Operational taxonomic unit at 2% sequence dissimilarity

a

Samples	Number of OTUs	Number of Sequences Observed Once	bc-chao-1	Good's coverage	Shannon	Simpson
L1	10,320	5,651	20,659	0.7	4.53	0.025
L2	4,788	2,111	9,872	0.8	4.51	0.024
L3	17,180	11,320	54,645	0.65	4.38	0.026
L4	13,785	6,241	23,377	0.8	4.48	0.026

b

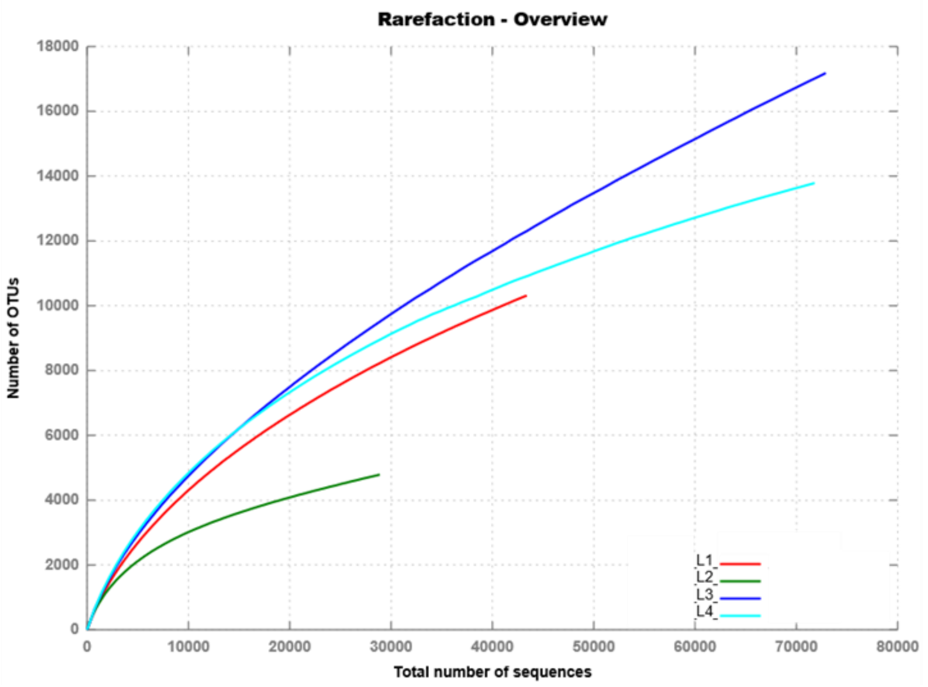

Figure S2. Alpha diversity a) alpha diversity table reporting the number of OTUs, Number of sequences observed once (singletons), bc-chao1 (index Bias-corrected Chao1 species richness estimator), good coverage per sample, Shannon-Wiener Index, and Simpson's evenness Index; b) rarefaction curves.

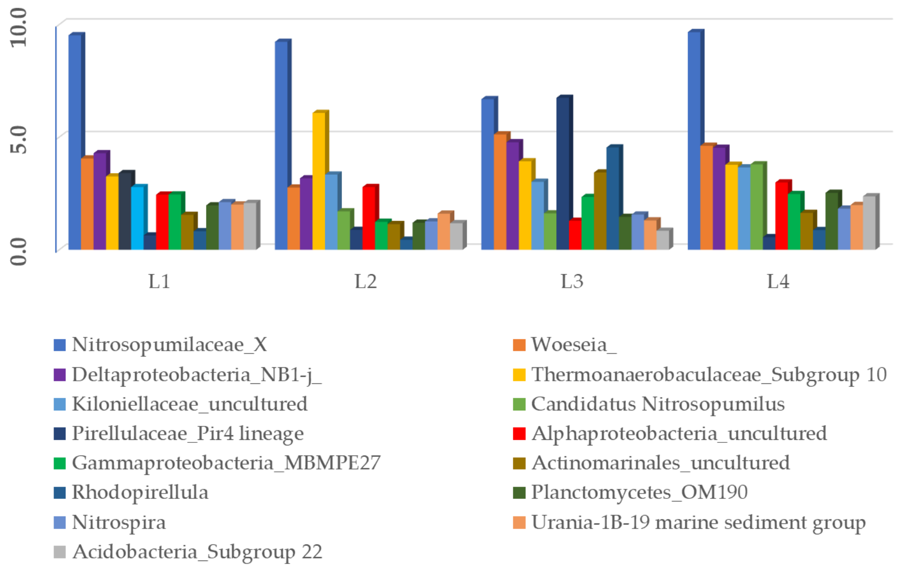

Figure S3. Top 15 genera detected in DSS prokaryotic community.

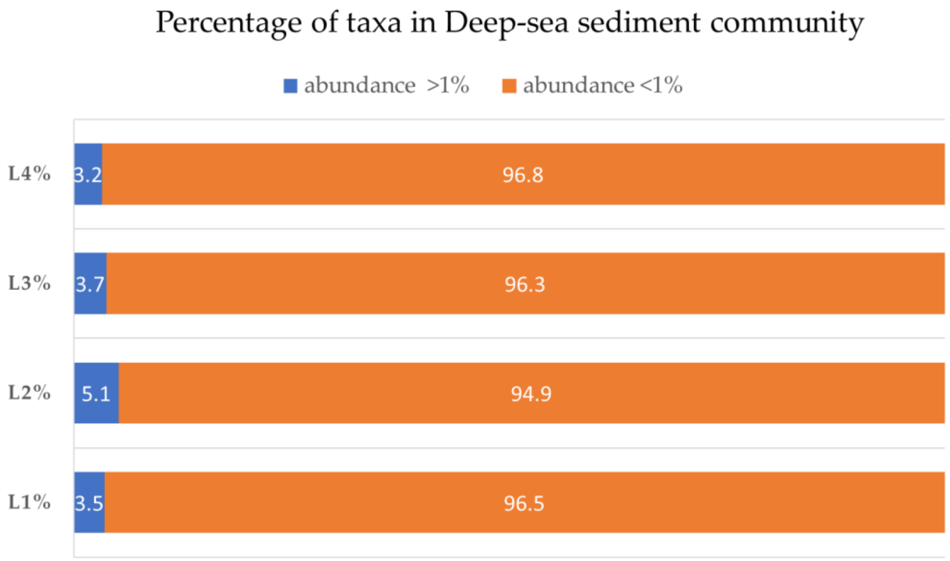

Figure S4. Percentage of taxa detected in Deep-sea sediment community. In blue is reported the percentage of taxa with an abundance >1%; in orange is reported the percentage of taxa with an abundance <1%.
